# Functional association between NUCKS1 gene and Parkinson disease: A potential susceptibility biomarker

**DOI:** 10.6026/97320630015548

**Published:** 2019-09-05

**Authors:** Sarita Singh, Prahlad Kishore Seth

**Affiliations:** 1 Biotech Park, Sector-G Jankipram, Kursi Road, Lucknow, India

**Keywords:** NUCKS1, Parkinson, rs823093, GWAS, SNP

## Abstract

Several Genome Wide Association Studies (GWASs) have reported that PARK16 gene locus possibly regulate the risk of Parkinson’s disease (PD). It contains functionally interesting candidate genes for PD, regulated by number of SNPs. In present study rs823093 polymorphism in NUCKS1 gene has been evaluated as significant performer in PD though its mechanism is not yet known. Here various regulatory and functional analyses were performed using computational tools and information from databases. The rs823093 variant was predicted to locate in enhancer histone marks in blood and have strong transcription in various parts of brain, heart, kidney and liver. PhenoScanner (a database of human genotype-phenotype associations) identified significant associations of this variant with many other diseases and phenotypic conditions as well. Gene expression analysis shows significant association with multiple human tissues and multiple genes together with NUCKS1. Further, the post mortem brain samples showed diverse expressions of NUCKS1 gene in PD patients compared to healthy samples. Besides, the metabolite analysis shows significant association with serotonin a known neurotransmitter, and other 15 metabolites. In addition, NUCKS1 also showed co-expression with ZNF43 and PLIN1 genes involved in cell cycle regulation presume their association in PD. Thus, these data links NUCKS1 gene as a potential disease susceptibility biomarker for PD.

## Background

Parkinson's disease (PD) is the second most common neurodegenerative disorder after Alzheimer's disease and is reported to affect up to 1 million Americans over the age 55 and up to 10 million individuals worldwide [1]. In India, with an aging population and increased life expectancy, it is expected that the disease burden due to PD will be enormous, but there is no prospective study to estimate its incidence and mortality. The incidence rates (IRs) in different countries vary from 1.5 to 20 per 100,000 per year [2]. The disease is hallmarked by degeneration of a neurons specifically dopaminergic neurons between the substantianigra (SN) and the striatum. Investigators have reported that a significant number of dopamine producing cells are lost in the substantianigra of PD patients [3]. As these neurons are destroyed, the clinical signs which characterize PD such as the slowed movements, rigidity and tremors start to appear. Another key neuropathological mark of PD is the formation of Lewy bodies, which are cytoplasmic inclusions primarily, composed of the α- synuclein protein. Lewy bodies have been reported in the dopaminergic neurons and other brain regions like the cortex and magnocellular basal forebrain nuclei [3].

Besides the above-mentioned causes, interactions between genetic and environmental factors seem to play a critical role in the development of PD [4]. Several candidate genes and susceptibility loci causing monogenic familial forms of PD have been identified during number of genome wide association studies [5]. PARK16 locus, located on chromosome 1q32, having five candidate genes i.e. NUCKS1, RAB7L1, SLC41A1, SLA45A3 and PM20D1, is significantly associated with PD [6]. NUCKS1 gene encodes a nuclear protein, 27 kD Nuclear casein kinase and cyclin-dependent kinase substrate 1. The conserved regions of NUCKS1 contain several consensus phosphorylation sites for casein kinase II (CK2) and cyclin-dependent kinases (Cdk) and a basic DNA-binding domain. NUCKS1 is similar to the high mobility group (HMG) family, which dominates chromatin remodeling and regulates gene transcription [7]. NUCKS1 plays a significant role in various diseases as susceptibility or potential marker and involve in several regulatory mechanisms [8-12] Noticeably, NUCKS1 involves in cell growth and proliferation as well as in DNA repair [13]. Using genome wide Association Studies (GWAS) it was found that NUCKS1 is a susceptibility gene for many diseases and a single disease can be associated with multiple SNPs of NUCKS1. However, the same SNP of NUCKS1 for same disease when examined in different races showed that NUCKS1 has diverse expression. Besides, NUCKS1 genotypes exhibit distinct expression for certain diseases. Though exact roles of NUCKS1 in diseases remain unclear, a significant association of expression and transcription levels of NUCKS1 with PD has been observed [14,15]. The rs823093 variant is located in the intron of NUCKS1 gene. The mechanism by which rs823093 variant affects the PD pathogenesis is not yet known. In the present study, using bioinformatics approaches an attempt has been made to examine the functional association of rs823093 polymorphism and PD, with an aim to identify a susceptibility biomarker.

## Methodology

### Regulatory and functional analysis

Regulatory and functional analysis of rs823093 variant was done using RegulomeDB database and HaploReg v4 tool, respectively. RegulomeDB could annotate genetic variants with known and predicted regulatory DNA elements which included regions of DNAase hypersensitivity, binding sites of transcription factors, promoter regions and binding motifs that play significant role in transcription regulation [16]. These datasets were collected from Gene Expression Omnibus (GEO), the Encyclopedia of DNA Elements (ENCODE) project, and published literature. HaploReg tool was used to annotate the non-coding variants which included information from the 1000 Genomes Project, chromatin state and protein binding annotation from the Roadmap Epigenomics and ENCODE projects, sequence conservation across mammals, the effect of SNPs on regulatory motifs, and expression of genes [17,18].

### Functional analysis using Genome Wide Association Study (GWAS)

Functional analysis using Genome wide association study (GWAS) of rs823093 variant was done using PhenoScanner package. This package included publicly available large-scale GWAS data, about 3 billion associations and over 10 million unique single nucleotide polymorphisms (SNPs) and a comprehensive phenotypes data [19]. In the present study, three kinds of functional analyses including the GWAS of diseases, metabolites, and gene expression analysis were performed. The PhenoScanner included 88 GWAS datasets with 76 kinds of diseases or phenotypes to carry out a GWAS analysis [20]. To perform metabolites analysis, PhenoScanner consisted of two metabolomics datasets [21,22]. For gene expression analysis, PhenoScanner included several datasets i.e. Geuvadis, GTEx (version 6), MuTHER, BIOSQTL, BLUEPRINT and Framingham etc.

### Validation of NUCKS1 gene expression in Parkinson's disease

Whole genome expression profiles in Parkinson's disease were analyzed to identify the responsible genes associated with Parkinson's disease. Here microarray expression data of total 25 samples (including 16 biopsy specimens of Parkinson's disease patients, and 9 healthy) from substantia nigra of postmortem human brain of Parkinson's disease patients was used [23]. A web tool GEO2R [24] to evaluate whether NUCKS1 gene is significantly deregulated in diseased cases compared with healthy samples at P < 0.01 significance level was used. Additionally, GeneMANIA tool [25] in Cytoscape4.0 package was used to study the correlation between expressed genes associated with rs823093 variant.

### Chart for Methodology:

**Figure 1:**
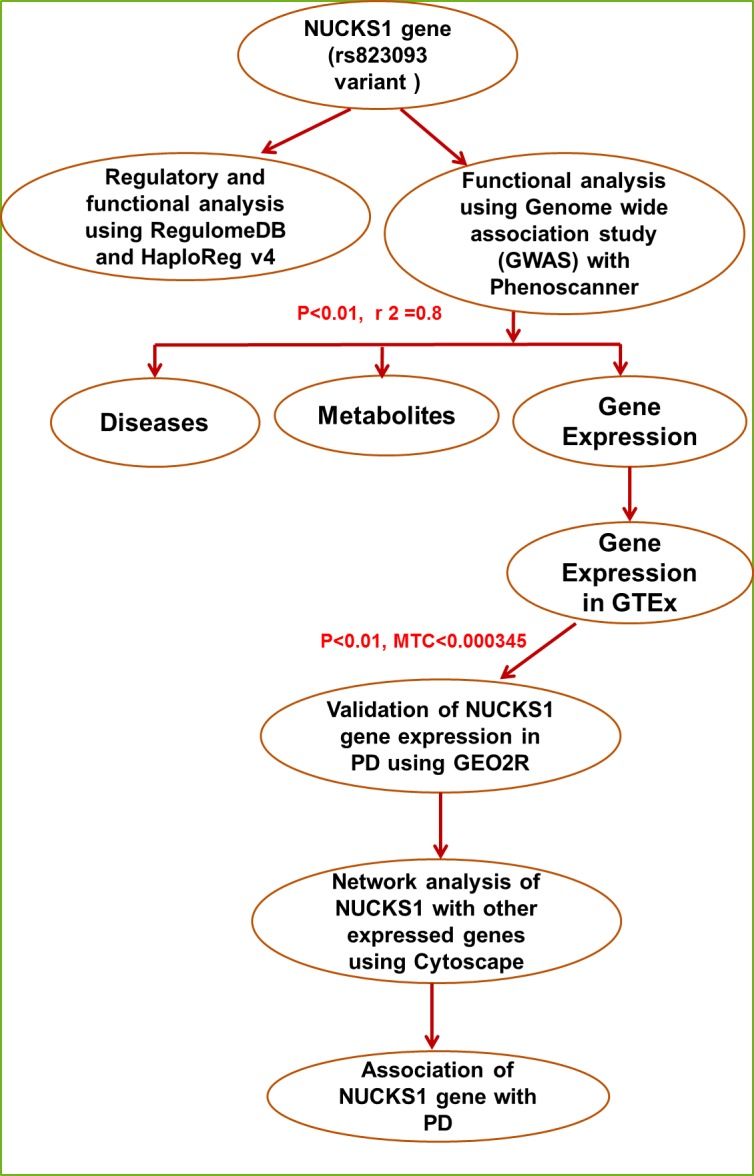
Flowchart for methodology

Flowchart of complete methodology is shown in Figure 1.

## Results

### Regulatory and functional analysis of rs823093 variant

Regulatory analysis of rs823093 variant shows score 6 in RegulomeDB, explaining that variant have binding motif i.e. OTX2 studies using Positional weight matrices (PWM) method. The histone modification study showed that the rs823093 variant is located in enhancer histone marks in blood and strong transcription in various parts of brain, heart, kidney and liver (Table 1 ). Functional analysis of rs823093 variant using HaploReg tool also shows enhancer histone marks in blood and six altered regulatory motifs i.e. DMBX1, FOXP1, LHX3, GSC, HMBOX1 and OBOX3. First three motifs are involves in brain related proteins.

### Association between rs823093 variant and type of disease or phenotype

GWAS of rs823093 variant identified thirty-eight significant associations at P<0.01. The variant is also significantly associated with other diseases or phenotypes besides Parkinson's disease such as Plateletcrit, Mucinous ovarian cancer, Chronic kidney disease, Particulate matter-associated QT prolongation, Monocyte percentage of white cells, Ulcerative colitis, Late onset Alzheimers disease, Neuroticism, Hip or knee osteoarthritis, Sporadic CreutzfeldtJakob disease and Inflammatory bowel disease etc. (details given in Table 2).

### Association between rs823093 variant and gene expression

The rs823093 variant shows fifty-three significant associations with gene expression at P<0.01. The analysis predicted significant associations of rs823093 variant with gene expression in multiple human tissues like brain, pancreas, thyroid, cells transformed fibroblasts, colon sigmoid, heart left ventricle, liver, lung, skin, small intestine, stomach, and whole blood, as shown in Table 3. These expressed genes include PM20D1, RAB7L1, NUCKS1, SLC41A1, CFHR2, PLIN1, KRBA2, ZNF43, CBX1, PGA4, FGD1, NFASC, SERPINB11, SLC1A7, TMEM54, CCDC28A, SLC45A3 and MFSD4. Significantly, rs823093 variant marks the expression of NUCKS1 gene in blood with P value 5.19e-09, 3.62e-05 and 3.41e-06 and in brain frontal cortex 3.11e-04.

### Association between rs823093 variant and metabolites

Sixteen metabolites showed remarkable associations with rs823093 variant at P<0.01 such as Glycerol Isobutyrylcarnitine, Pantothenate, Lactate, 4-acetamidobutanoate, LDL, Serotonin (5HT), Gamma-glutamylleucine, Phosphate, Cholesterol, Caprylate, Oleate, Heptanoate, 7-methylguanine, Glucose and Nacetylornithine as listed in Table 4.

### Validation of NUCKS1 gene expression in Parkinson's disease

**Figure 2:**
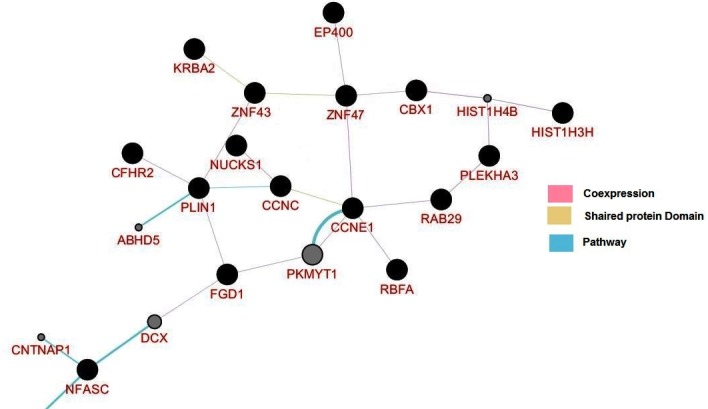
Correlation between NUCKS1 with other expressed genes

The expression of NUCKS1 gene in Parkinson's disease has been evaluated using GEO2R tool. Seven probes i.e. 226880_at, 223661_at, 229353_s_at, 224582_s_at, 217802_s_at, 224581_s_at and 222424_s_at were identified in the expression of NUCKS1 gene in gene expression profile of substantianigra of postmortem brain from Parkinson's disease patients. Each probe represent different region of NUCKS1 gene and may have same or different transcript. All these probes are found to be deregulated in Parkinson's cases compared with healthy individuals. Among these 226880_at is significantly deregulated with P = 2.99e-04 and log2 (fold change) = -0.861(Table 5). There are 18 different genes, which are regulated by rs823093 as described in Table 3. Simultaneously, evaluation of expression of other 17 genes with NUCKS1 revealed that three of them are also have different expression in Parkinson's cases i.e. RAB7L1, NFASC and MFSD4 at P<0.01 and multiple testing correction (MTC) threshold 0.000345 (Table 5). Further, network analysis of NUCKS1 gene with other expressed genes regulated by rs823093 variant, revealed that NUCKS1 is co-expressed with ZNF43 and PLIN1genes and ZNF43 shared a protein domain with KRBA2 gene (Figure 2).

## Discussion

Genome-wide association studies have shown that PARK16 locus has significant association with Parkinson's disease [26,27]. NUCKS1 is also reported as one of the important gene at PARK16 locus and has noteworthy association with PD [16]. NUCKS1 encodes a nuclear protein including phosphorylation sites for casein kinase 2 and cyclin-dependent kinases substrate. It is vertebrate specific gene ubiquities in the brain and peripheral tissues [29].The casein kinase 2 has been reported to be involved in altering the dopamine signaling as well as hyper phosphorylation of alpha-synuclein [30,31] and cyclin-dependent kinases, suppress dopamine D1 signaling in the striatum by phosphorylation of postsynaptic protein DARPP-32 [32]. Some previous studies have also reported that cell-cycle protein mediates the degeneration of dopaminergic neurons [7,33]. An earlier study reported that rs823128 variant of NUCKS1 might affect PD risk by altering the transcription factor-binding capability of the genes [34] and also reported as hub gene in a gene network analysis study on Parkinson's disease [35].

Hence, NUCKS1 may be crucial for cell cycle progression. Though a definite mechanism of NUCKS1 in PD is not known, it may presumably be involved in the pathogenesis of PD. A complete functional analysis of rs823093 variant of NUCKS1 gene predicated a score of 6, on application of RegulomeDB, using PWM method, suggesting that rs823093 is likely to affect OTX2 motif (chr1: 205689224 - 205689231b) and is associated with histone modification in blood and strong transcription in various parts of brain besides, heart, kidney and liver. Application of HaploReg (version 4.1), also suggested that rs823093 is associated with enhancement of histone modification in blood verifying the findings in HaploReg (version 4.1). PhenoScanner GWAS analysis, showed that rs823093 is not only associated with Parkinson disease, but also is significantly associated with other diseases or phenotypes including Plateletcrit, Mucinous ovarian cancer, Chronic kidney disease, ovarian cancer, Ulcerative colitis, Late onset Alzheimers disease and many other. A study on Alzheimer's disease in Han chinese population also suggested that Parkinson's disease GWAS-Linked loci i.e. RAB7L1-NUCKS1 is associated with late Alzheimer's disease [36]. The rs823114 variant of NUCKS1 also indicates decreased risk of susceptibility to PD in Han Chinese male and association of three candidate genetic variants in RAB7L1/NUCKS1, MCCC1 and STK39 with sporadic Parkinson's disease [37,38].It is interesting that GA haplotype is reported as risk factor for PD and phenoscanner testify that rs823093 is associated with GA haplotype [39].

PhenoScanner gene expression analysis showed that rs823093 is significantly associated with expression of multiple genes in multiple human tissues together with NUCKS1. Also rs823093 was identified to be expressively associated with other 16 metabolites including Serotonin (5HT) using PhenoScanner metabolites option.

**Figure 3:**
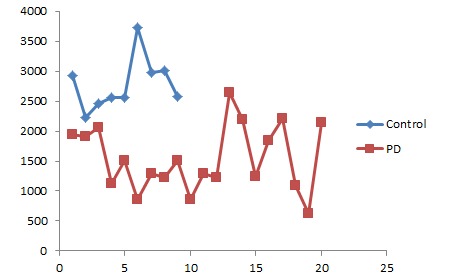
Expression graph of NUCKS1 gene: PD Vs. Control

Interestingly serotogenic dysfunction has a direct relevance to Parkinson's disease non-motor symptoms, like depression, fatigue, weight changes, and visual hallucinations [40]. Substatianigra of postmortem human brain exhibited different expression of NUCKS1 gene in PD patients as compared with healthy samples (PD = 16; Healthy = 09), likewise the gene expression graph (Figure 3) for 226880_at probe depicts that NUCKS1 is down regulated in PD patients. Additionally, NUCKS1 is co-expressed with ZNF43 and PLIN1 genes where ZNF43 share a protein domain with KRBA2, in network analysis. Therefore it is presume that these three genes may also works as susceptible gene for PD pathogenesis but more study has to be needed.

## Conclusion

NUCKS1 is reported as one of the significant gene at PARK16 locus and has remarkable connotation with PD but its mechanism is not yet known. In current study a comprehensive functional analysis of rs823093 variant of NUCKS1 gene has been done using gene expression, disease association, network and metabolite analysis. The findings of stated analysis verified the possible association of NUCKS1 gene with PD, which may serve as susceptibility marker for PD.

## Figures and Tables

**Table 1:** Histone modification analysis

Location	Chromatin State	Tissue
chr1:205689200..205690000	Genic enhancers	Blood
chr1:205684600..205691400	Strong transcription	Fetal Brain Female
chr1:205685400..205690800	Strong transcription	Fetal Brain Male
chr1:205686600..205690200	Strong transcription	Brain Inferior Temporal Lobe
chr1:205688000..205690600	Strong transcription	Brain Substantia Nigra
chr1:205689000..205692200	Strong transcription	Brain Hippocampus Middle
chr1:205682800..205711200	Strong transcription	Fetal Thymus
chr1:205683400..205693800	Strong transcription	Fetal Adrenal Gland
chr1:205684600..205693000	Strong transcription	Fetal Kidney
chr1:205688000..205690000	Strong transcription	Fetal Heart
chr1:205688000..205690200	Strong transcription	Right Ventricle
chr1:205688000..205690400	Strong transcription	Sigmoid Colon
chr1:205688400..205693200	Strong transcription	Liver
chr1:205680400..205694200	Quiescent/Low	Right Atrium

**Table 2:** Association between rs823093 and type of disease or phenotype

Disease or phenotype	PMID	P-value	No. of samples
Prostate specific antigen levels	25434496	5.00E-13	NA
Parkinson's disease	22438815	1.38E-11	4258
Parkinson's disease	19915576	4.88E-09	3509
Parkinson's disease	19915575	7.29E-08	5691
Parkinson's disease	21248740	7.29E-08	2796
Parkinson's disease	25064009	2.22E-06	108990
Plateletcrit	27863252	2.58E-04	173480
Parkinson's disease	21738487	1.90E-04	33050
HbA1c	28898252	1.19E-03	123665
Body mass index	28892062	9.80E-04	173430
Body mass index in males greater than 50 years of age	26426971	1.70E-03	92442
Body mass index females	28892062	3.95E-04	82438
Mucinous ovarian cancer	28346442	2.60E-03	42090
Chronic kidney disease	26831199	3.30E-03	117165
Childhood BMI	26604143	3.55E-03	35669
Chronic kidney disease	20383146	3.90E-03	62237
Insulin sensitivity index adjusted for BMI interaction	27416945	4.10E-03	16753
Particulate matter-associated QT prolongation	28749367	4.56E-03	22158
Urea	28887542	4.66E-03	9961
ovarian cancer	28346442	5.05E-03	42895
Monocyte percentage of white cells	27863252	5.24E-03	173480
Ulcerative colitis	26192919	5.30E-03	27432
Body mass index adjusted for physical activity in males	28448500	5.85E-03	84503
Testosterone	28887542	6.06E-03	4387
Body mass index	25673413	6.22E-03	339224
Late onset Alzheimer’s disease	21390209	6.36E-03	3595
Neuroticism	27089181	6.44E-03	170911
Hip or knee osteoarthritis	22763110	6.74E-03	18419
Waist circumference in female smokers	28443625	7.42E-03	20595
Body mass index in physically inactive individuals	28448500	8.00E-03	42066
Diabetic nephropathy	16775037	8.01E-03	1795
Body mass index adjusted for smoking in males	28443625	8.38E-03	102746
Body mass index in physically inactive individuals	28448500	9.08E-03	46393
Sporadic Creutzfeldt Jakob disease	22210626	9.23E-03	7872
Inflammatory bowel disease	26192919	9.42E-03	34652
Granulocyte percentage of myeloid white cells	27863252	9.58E-03	173480
Body mass index in male non-smokers	28443625	9.88E-03	78101
Albumin	28887542	9.93E-03	9961

**Table 3:** Association between rs823093 and gene expression

Gene	Tissue	No. of sample	Beta	SE	P-value	PMID	Source
PM20D1	Adipose subcutaneous	385	0.4991	0.1489	9.02E-04	25954001	GTEx
PM20D1	Testis	225	0.6166	0.1796	7.36E-04	25954001	GTEx
PM20D1	Whole blood	2116	NA	NA	1.22E-30	27918533	BIOSQTL
RAB7L1	Adipose subcutaneous	385	-0.3531	0.09168	1.42E-04	25954001	GTEx
RAB7L1	Artery tibial	388	-0.362	0.08334	1.89E-05	25954001	GTEx
RAB7L1	Brain anterior cingulate cortex BA24	109	-0.6235	0.1707	4.42E-04	25954001	GTEx
RAB7L1	Brain cortex	136	-0.4474	0.1162	1.96E-04	25954001	GTEx
RAB7L1	Breast mammary tissue	251	-0.3681	0.09399	1.23E-04	25954001	GTEx
RAB7L1	Esophagus mucosa	358	-0.2519	0.07573	9.94E-04	25954001	GTEx
RAB7L1	Esophagus muscularis	335	-0.3319	0.0768	2.14E-05	25954001	GTEx
RAB7L1	Heart left ventricle	272	-0.3227	0.08435	1.70E-04	25954001	GTEx
RAB7L1	Muscle skeletal	491	-0.346	0.06334	8.01E-08	25954001	GTEx
RAB7L1	Skin sun exposed lower leg	414	-0.3987	0.07704	3.86E-07	25954001	GTEx
RAB7L1	Thyroid	399	-0.3763	0.07258	3.77E-07	25954001	GTEx
RAB7L1	Monocytes	194	-0.9174	0.243	1.60E-04	27863251	BLUEPRINT
RAB7L1	Neutrophils	192	-0.8078	0.2442	9.42E-04	27863251	BLUEPRINT
RAB7L1	Adipose visceral omentum	313	-0.3261	0.07763	3.65E-05	25954001	GTEx
RAB7L1	Artery aorta	267	-0.4965	0.1201	5.11E-05	25954001	GTEx
RAB7L1	Brain hippocampus	111	-0.9282	0.1895	4.29E-06	25954001	GTEx
RAB7L1	Colon sigmoid	203	-0.4021	0.1047	1.75E-04	25954001	GTEx
RAB7L1	Esophagus gastroesopha-geal junction	213	-0.3007	0.07885	1.89E-04	25954001	GTEx
RAB7L1	Heart atrial appendage	264	-0.3142	0.0907	6.44E-04	25954001	GTEx
RAB7L1	Nerve tibial	361	-0.2752	0.07424	2.50E-04	25954001	GTEx
RAB7L1	Pancreas	220	-0.4918	0.1111	1.63E-05	25954001	GTEx
RAB7L1	Brain hypothalamus	108	-0.9381	0.2511	3.35E-04	25954001	GTEx
RAB7L1	Stomach	237	-0.4187	0.1214	6.84E-04	25954001	GTEx
RAB7L1	Whole blood	2116	NA	NA	1.35E-21	27918533	BIOSQTL
RAB7L1	Lung	383	-0.3165	0.08726	3.35E-04	25954001	GTEx
NUCKS1	Brain frontal cortex BA9	118	-0.5593	0.1495	3.11E-04	25954001	GTEx
NUCKS1	Prostate	132	0.4927	0.1391	4.28E-04	25954001	GTEx
NUCKS1	Whole blood	2116	NA	NA	5.19E-09	27918533	BIOSQTL
NUCKS1	Peripheral blood	5311	NA	NA	3.41E-06	24013639	Westra-H
NUCKS1	Lung	278	0.2174	0.06255	6.05E-04	25954001	GTEx
NUCKS1	Whole blood	369	0.1994	0.04756	3.62E-05	25954001	GTEx
SLC41A1	Thyroid	278	-0.3251	0.09241	5.21E-04	25954001	GTEx
SLC41A1	Thyroid	399	-0.2909	0.06974	3.88E-05	25954001	GTEx
SLC41A1	Lymphoblasto-id cell lines	462	NA	NA	3.53E-08	24037378	Geuvadis
SLC41A1	Brain anterior cingulate cortex BA24	109	-0.467	0.1296	5.21E-04	25954001	GTEx
CFHR2	Peripheral blood monocytes	1490	NA	NA	4.47E-06	20502693	Zeller
PLIN1	Whole blood	5257	-0.0209	0.00473	1.12E-05	28122634	Framingham
KRBA2	Whole blood	5257	NA	NA	2.69E-05	28122634	Framingham
ZNF43	Whole blood	5257	0.0435	0.01073	5.13E-05	28122634	Framingham
CBX1	Whole blood	5257	0.0352	0.00876	5.95E-05	28122634	Framingham
PGA4	Whole blood	5257	-0.042	0.01047	6.12E-05	28122634	Framingham
FGD1	Whole blood	5257	0.013	0.00326	6.81E-05	28122634	Framingham
NFASC	Prostate	87	0.7023	0.1901	4.49E-04	25954001	GTEx
NFASC	Pituitary	87	-0.5927	0.1395	6.98E-05	25954001	GTEx
SERPINB11	Whole blood	5257	-0.0322	0.00813	7.55E-05	28122634	Framingham
SLC1A7	Whole blood	5257	-0.0192	0.00485	7.56E-05	28122634	Framingham
TMEM54	Whole blood	5257	-0.0253	0.00644	8.55E-05	28122634	Framingham
CCDC28A	Whole blood	5257	0.0227	0.00583	9.89E-05	28122634	Framingham
SLC45A3	Adipose subcutaneous	385	0.2693	0.07622	4.72E-04	25954001	GTEx
MFSD4	Cells transformed fibroblasts	272	0.585	0.1505	7.32E-04	25954001	GTEx

**Table 4:** Association between rs823093 and metabolites

Metabolite	No. of sample	Beta	SE	P-value	PMID
Glycerol	1735	NA	NA	1.60E-04	21886157
Isobutyrylcarnitine	1725	NA	NA	3.10E-04	21886157
Pantothenate	911	NA	NA	3.20E-04	21886157
Lactate	24871	0.06257	0.02158	4.65E-03	27005778
4-acetamidobutanoate	6523	0.016	0.0057	4.92E-03	24816252
LDL	19273	0.05838	0.02064	4.99E-03	27005778
Serotonin (5HT)	5791	0.0281	0.0106	5.04E-03	24816252
Gamma-glutamylleucine	7354	0.0139	0.005	5.04E-03	24816252
Phosphate	7341	-0.0113	0.0042	7.04E-03	24816252
Cholesterol	7365	-0.0127	0.0048	7.41E-03	24816252
Caprylate	7355	-0.0154	0.0058	7.43E-03	24816252
Oleate	7323	-0.0178	0.0067	7.58E-03	24816252
Heptanoate	7353	-0.0295	0.0112	8.19E-03	24816252
7-methylguanine	5804	0.0349	0.0133	8.45E-03	24816252
Glucose	7325	-0.0099	0.0038	9.16E-03	24816252
N-acetylornithine	7146	0.0326	0.0126	9.49E-03	24816252

**Table 5:** Expression analysis of genes including NUCKS1, regulated by rs823093 variant in Parkinson's disease dataset

Probe ID	Gene	t-statistics	Fold change (log2)	P-value
239929_at	PM20D1	1.08838876	0.37	2.87E-01
218700_s_at	RAB7L1	2.19856388	0.467	3.72E-02
218699_at	RAB7L1	2.12573885	0.397	4.34E-02
243777_at	RAB7L1	-1.36547912	-0.572	1.84E-01
226880_at	NUCKS1	-4.18256211	-0.861	2.99E-04
223661_at	NUCKS1	-2.09624443	-0.654	4.61E-02
224582_s_at	NUCKS1	-1.72103478	-0.316	9.74E-02
224581_s_at	NUCKS1	-1.2063773	-0.178	2.39E-01
217802_s_at	NUCKS1	-1.03533831	-0.183	3.10E-01
222424_s_at	NUCKS1	-0.775199	-0.142	4.45E-01
229353_s_at	NUCKS1	-0.0254664	-0.567	9.80E-01
225570_at	SLC41A1	-1.88066702	-0.25	7.15E-02
206910_x_at	CFHR2	1.40070154	0.268	1.73E-01
205913_at	PLIN1	-0.49919732	-0.287	6.22E-01
1558533_at	KRBA2	0.71072732	0.187	4.84E-01
206695_x_at	ZNF43	-1.79511489	-0.289	8.45E-02
222136_x_at	ZNF43	-0.67058716	-0.111	5.09E-01
201518_at	CBX1	0.77565557	0.832	4.45E-01
213265_at	PGA4	0.22672692	0.965	8.22E-01
204819_at	FGD1	1.7057522	0.364	1.00E-01
213438_at	NFASC	-0.97811674	-0.189	3.37E-01
243645_at	NFASC	-0.87218298	-0.304	3.91E-01
1552463_at	SERPINB11	0.99260234	0.435	3.30E-01
210923_at	SLC1A7	-0.72614289	-0.288	4.74E-01
225536_at	TMEM54	-0.86172346	-0.163	3.97E-01
209479_at	CCDC28A	-1.74253691	-0.345	9.35E-02
228696_at	SLC45A3	0.5812718	0.124	5.66E-01
242372_s_at	MFSD4	-1.11253201	-0.808	2.76E-01
238862_at	MFSD4	-0.43285076	-0.188	6.69E-01
